# Author Correction: Evolution and universality of two-stage Kondo effect in single manganese phthalocyanine molecule transistors

**DOI:** 10.1038/s41467-022-28154-6

**Published:** 2022-01-28

**Authors:** Xiao Guo, Qiuhao Zhu, Liyan Zhou, Wei Yu, Wengang Lu, Wenjie Liang

**Affiliations:** 1 Songshan Lake Materials Laboratory, Dongguan, Guangdong, China; 2 Beijing National center for Condensed Matter Physics, Beijing Key Laboratory for Nanomaterials and Nanodevices, Institute of Physics, Chinese Academy of Sciences, Beijing, P.R. China; 3 CAS Center of Excellence in Topological Quantum Computation and School of Physical Sciences, University of Chinese Academy of Sciences, Beijing, P.R. China

**Keywords:** Electron transfer, Nanoscale devices, Electronic structure of atoms and molecules, Exotic atoms and molecules, Quantum information

Correction to: *Nature Communications* 10.1038/s41467-021-21492-x, published online 10 March 2021.

The original version of this Article contained an error in Ref. 34, which was an incorrect entry. The correct form of Ref. 34 is:

Karki, D. B., Mora, C., von Delft, J. & Kiselev, M. N. Two-color Fermi-liquid theory for transport through a multilevel Kondo impurity. *Phys. Rev. B* **97**, 195403 (2018).

The original version of this Article contained an error in Ref. 8, which was incorrectly given with the wrong article title as “effect in an integer-spin quantum dot”. The correct form of Ref. 8 is:

Sasaki, S., De Franceschi, S., Elzerman, J. M., van der Wiel, W. G., Eto, M., Tarucha, S. & Kouwenhoven, L. P. Kondo effect in an integer-spin quantum dot. *Nature* **405**, 764-767 (2000).

The original version of this Article contained an error in Ref. 10, which was incorrectly given with the wrong author list and article page range. The correct form of Ref. 10 is:

Jarillo-Herrero, P., Kong, J., van der Zant, H. S. J., Dekker, C., Kouwenhoven, L. P. & De Franceschi, S. Orbital Kondo effect in carbon nanotubes. *Nature* **434**, 484-488 (2005).

The original version of this Article contained an error in Ref. 21, which was incorrectly given with the wrong article title as “Kondo effect in a quantum dot at a high magnetic field”. The correct form of Ref. 21 is:

van der Wiel, W. G., De Franceschi, S., Elzerman, J. M., Tarucha, S., Kouwenhoven, L. P., Motohisa, J., Nakajima, F. & Fukui, T. Two-stage Kondo effect in a quantum dot at a high magnetic field. *Phys. Rev. Lett*. **88**, 126803 (2002).

All of these errors in references have been corrected in the PDF and HTML version of the Article.

