# Supplementary material for: Evolution and universality of two-stage Kondo effect in single manganese phthalocyanine molecule transistors
Source: Nat Commun. 2021 Mar 10;12:1566. doi: 10.1038/s41467-021-21492-x (PMC7946881; doi:10.1038/s41467-021-21492-x)
Supplement: Supplementary file 1 — Supplementary Information [file 41467_2021_21492_MOESM1_ESM.pdf]

SUPPLEMENTARY INFORMATION

Evolution and universality of Two-stage Kondo effect in single manganese phthalocynine molecule transistors

Xiao Guo[1,2,3], Qiuhao Zhu[2,3], Liyan Zhou[2,3], Wei Yu[2,3], Wengang Lu[2,3], Wenjie Liang[1,2,3]*

1 Songshan Lake Materials Laboratory, Dongguan, Guangdong 523808, China
2 Beijing National center for Condensed Matter Physics, Beijing Key Laboratory for Nanomaterials and Nanodevices, Institute of Physics, Chinese Academy of Sciences, Beijing, 100190, P. R. China;
3 CAS Center of Excellence in Topological Quantum Computation and School of Physical Sciences, University of Chinese Academy of Sciences, Beijing 100190, P.R. China.

*E–mail: wjliang@iphy.ac.cn

Supplementary Methods

1.Device fabrication

Single MnPc molecule transistors devices were fabricated by electromigration technique. A schematic diagram is shown in Fig. 1(a). A narrow gold nanowire was fabricated by electron-beam lithography on an aluminum pad with a ~3nm oxide layer serving as the gate electrode. Electrical current was applied to the gold nanowire to create two closely spaced electrodes with a gap about 1nm. To incorporate single molecules, dilute alcohol solution of MnPc was deposited on a chip containing the array of gold nanowires before the electromigration breaking. Immediately after breaking process, the chips are transferred to a cryostat and cooled down to base temperature to freeze electrodes and stabilize molecules.

2.Identification of single MnPc molecule.

Excited states that arise from the internal vibration of measured molecules could be used to identify unique molecular devices. Devices 1 and 2 show pronounced excited states at 63meV and 16meV respectively (Supplementary Fig.1), matching infrared spectra of metal-phthalocyanine derivatives[1-3] for out of plane vibration and in plane stretching modes. The excited conductance peaks should appear symmetrically in both directions of bias. However, due to the unsymmetrical coupling of molecules to source and drain electrodes in our devices, the excited peaks only appear on the one side of bias (Supplementary Fig.1 a and b). The dI/dV-V_{sd} spectrums of two devices are shown in Supplementary Fig.1 c and d, in which the steps features correspond to the inelastic cotunneling processes through vibration states of molecules. When the biases are greater than energy of vibration states, the spectrums become very noisy. This phenomenon appears quite often in our devices when the molecule and electrodes are strongly coupled, but the origin is not clear, probability arise from the interference of different conduction

channels.

The additional energies, i.e. the energies required to add one more electron to a molecule, are typically hundreds of meV for the single molecule junctions. The addition energy of our devices cannot be measured directly due to the instability of molecular junction under a high bias voltage. But from the measurement range in both V_{sd} and V_g, we estimate the lower bound of additional energy is 120meV for device1 and 160meV for device2, much larger than that of gold clusters occasionally formed during current breaking process. Observation of molecule specific vibration and large addition energy supports that measured signals come from electrons passing through individual MnPc molecule in the metal junction.

3.Characterization of spin 1/2 fully screened Kondo effect.

In blocking region I of both device 1 and 2, sharp zero-bias conductance peaks appear. The peak is strongly temperature dependent. Differential conductance (dI/dV) versus bias V_{sd} for various temperature at V_g=2V for device 1 is shown in Supplementary Fig. 2a. As temperature is raised, the peak height decreases in a logarithmic fashion [Supplementary Fig. 2b]. The single peak also splits linearly under magnet field, as shown in Supplementary Fig. 2c, in which g factor is equal to 1.79. These phenomena indicate this is a Kondo resonance. The temperature dependence of spin S=1/2 Kondo peak height follows an approximate scaling form[4]

$$G(T) = G_0/[1 + (2^{1/s} - 1)(T/T_{\mathrm{k},1/2})^2]^s + G_c \qquad (1)$$

where G_0 is the zero temperature conductance, G_c is a background conductance and $T_{k,1/2}$ is the Kondo temperature. We use this formula to fit Kondo peak height at different temperature (Supplementary Fig. 2b) and get $T_{K,1/2}$=3.21$\pm$0.02K. Supplementary Fig. 2b also shows the data fitting to numerical renormalization group(NRG) result for S=1 and 3/2 underscreened Kondo processes[5]. The data fit best to spin 1/2 model, excluding the underscreened Kondo effect for S=1 and 3/2. The detailed temperature dependences analysis of Kondo resonance shows this is a fully screened single channel spin 1/2 ground state instead of 3/2 ground state. These evidences clearly proves that MnPc has S=1/2 ground state in region I and the Kondo resonance we observed in this region is fully screened spin 1/2 Kondo process.

The Kondo temperature can be estimated from the magnet field measurement. The critical field associated with the onset of Zeeman splitting $B_c = 0.5k_B T_{K,1/2}/g\mu_B$, where μ_B is Bohr magneton. As shown in Supplementary Fig. 2c, the critical field $B_c \approx 3\mathrm{T}$, the corresponding $T_{K,1/2}$=3.32K. For S=1/2 single impurity model, the Kondo temperature can also be estimated from the peak width of the Kondo resonance by the formula[6], $\mathrm{FWHM} = 2\sqrt{(\pi k_B T)^2 + 2(k_B T_{k,1/2})^2}$, where k_B is the Boltzmann constant. At 300mK, FWHM is 662uV at V_g=2V in Supplementary Fig. 2a, $T_{k,1/2}$ is estimated to be 3.78K. All the estimations are consistent with the fitting result of temperature measurement.

Supplementary Figures

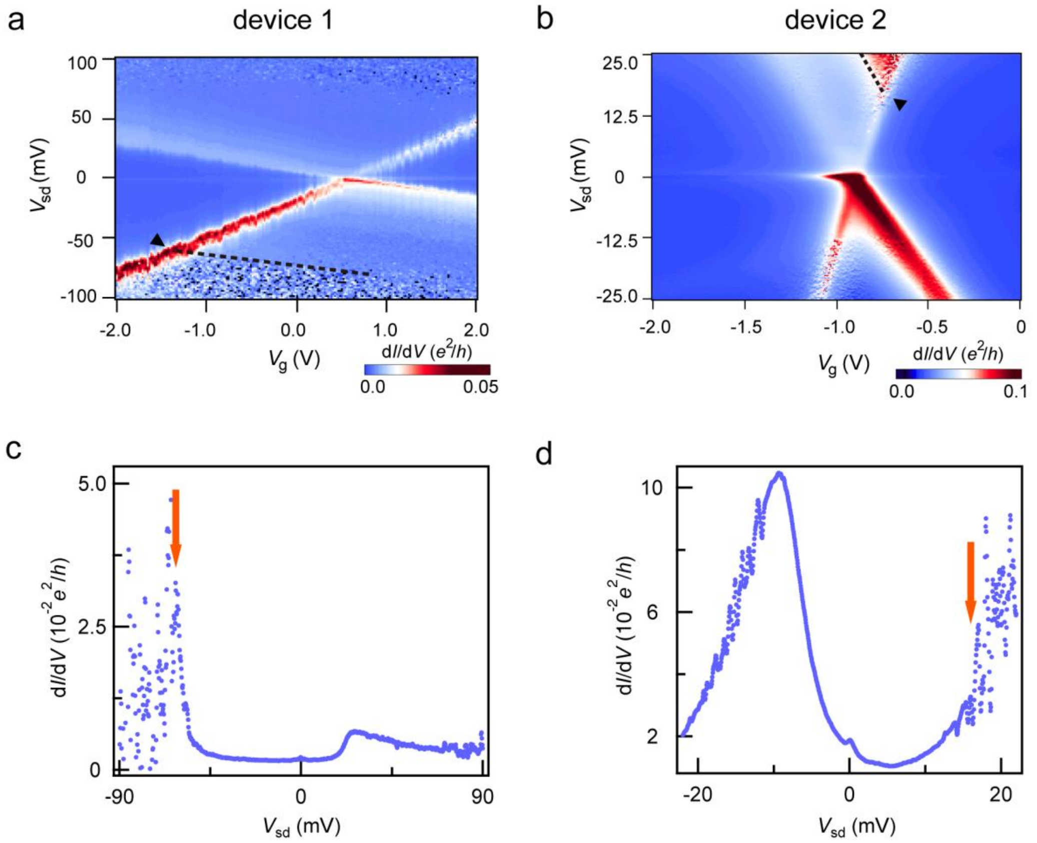

Supplementary Fig. 1. Excited levels of MnPc molecule. **a**,**b**. Differential conductance plots showing a larger bias-voltage range than those in main text for device1 (**a**), device2 (**b**). Excited levels arising from internal vibrational modes of MnPc molecule are marked by black dash line and arrows. **c**. Crosssection of the two-dimensional plot in **a** at V_g=-1.73V. The position of excited state is marked by an arrow. **d**. Same as **c** but for device 2 at V_g=-0.726V.

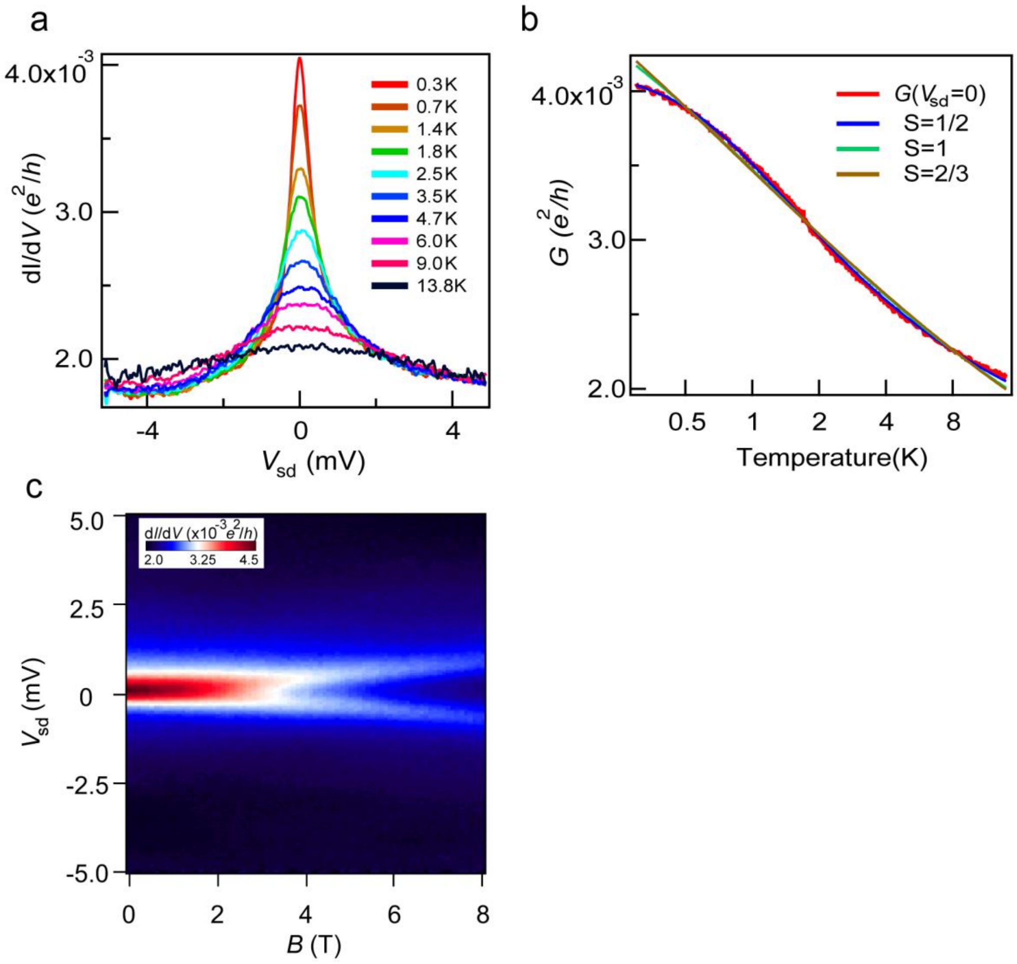

Supplementary Fig. 2. Spin 1/2 fully screened Kondo effect. **a**. Plot of differential conductance d*I*/d*V* versus V_{sd} at various temperatures (at V_g=2V for device 1). **b**. Plot of the Kondo peak height *G*(V_{sd}=0) in a as a function of temperature. The data is fitted to S=1/2, S=1 and S=2/3 model respectively. **c**. Differential conductance d*I*/d*V* for device 1 at V_g=1.6V as a function of V_{sd} and magnet field B.

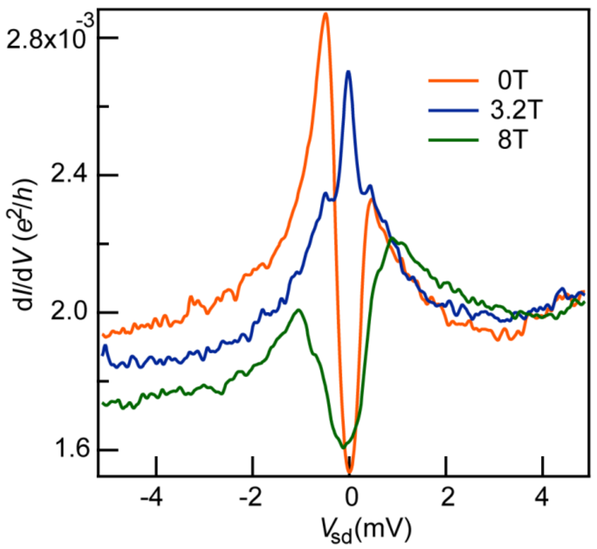

Supplementary Fig. 3 The differential conductance profiles at B=0T, 3.2T and 8T in Fig. 2c (V_g=-2V for device 1). B_0=3.2T is the critical point. The peak at B=3.2T is not greater than the peaks at B=0, indicating only one channel participates.

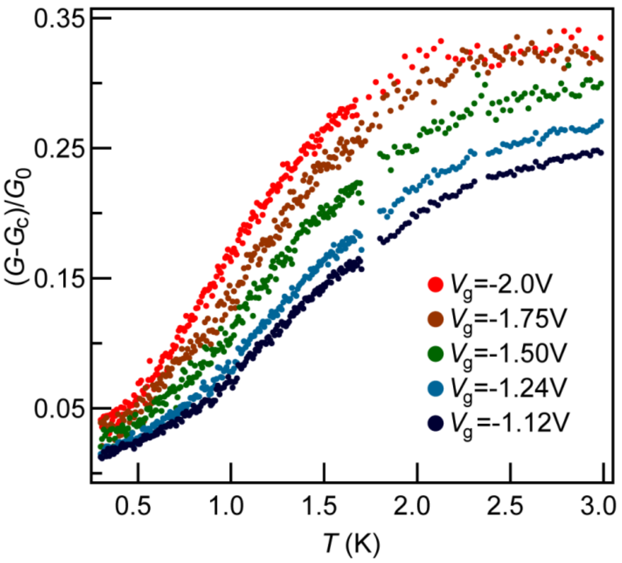

Supplementary Fig. 4 Plot scaled zero bias conductance $(G\text{-}G_c)/G_0$ at different gate voltage as a function of temperature. The temperature dependence of profiles at different gate voltage is different with each other, indicating T^* is gate dependent.

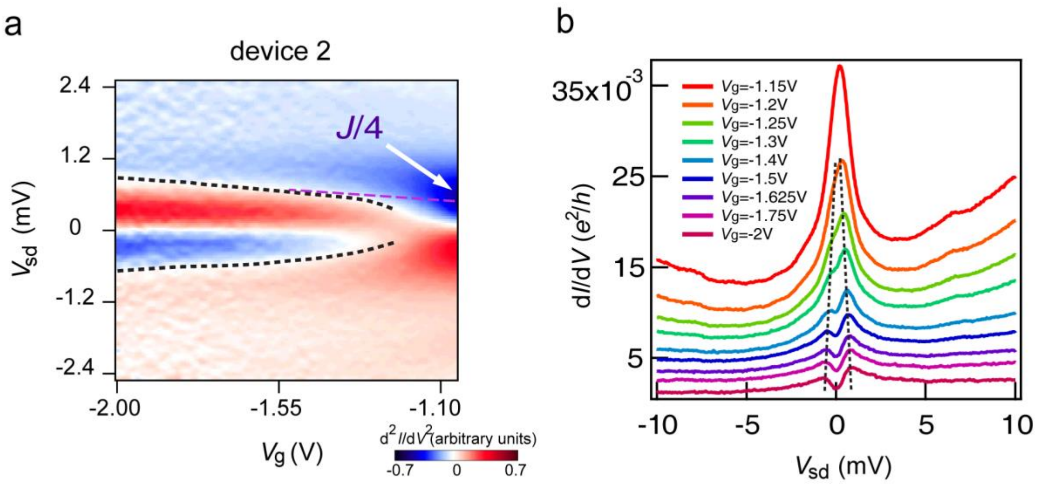

Supplementary Fig. 5 Gate dependent singlet-triplet energy splitting in device 2. **a**, Color plot of numerical derivative of d*I*/d*V* as a function V_{sd} and V_g in region II for device 2. The black dash line indicates the peak position. Far away from singlet-triplet transition point, the peak position varies almost linearly with gate voltage. When approaching transition point, the splitting deviate from linear relation, indicating a ferromagnetic coupling between two electrons (J<0). By extending the linear peak position to the gate voltage when transition should happen, we can estimate the value of J/4, about -0.4meV, which is quantitatively in agreement with device 1. **b**, Plot of d*I*/d*V* against V_{sd} in region II of device 2 at different gate voltage. The peak positions are marked by dash line. The curves are shifted vertically for clarification.

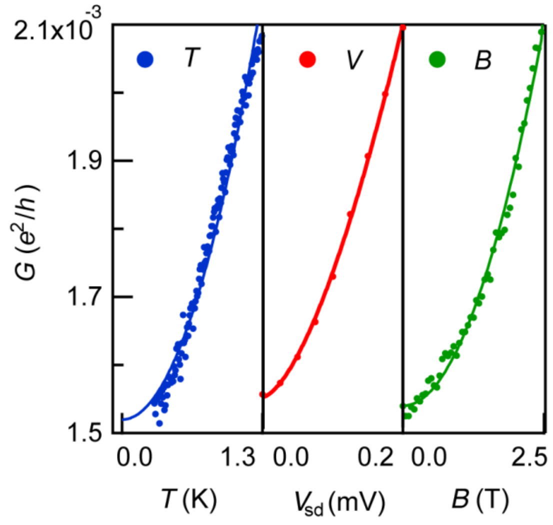

Supplementary Fig. 6 The low-energy differential conductance(V_g=-2V) fitted to a power law with *T*(temperature), *V*(bias) and *B*(magnetic field). The solid lines are power-fits.

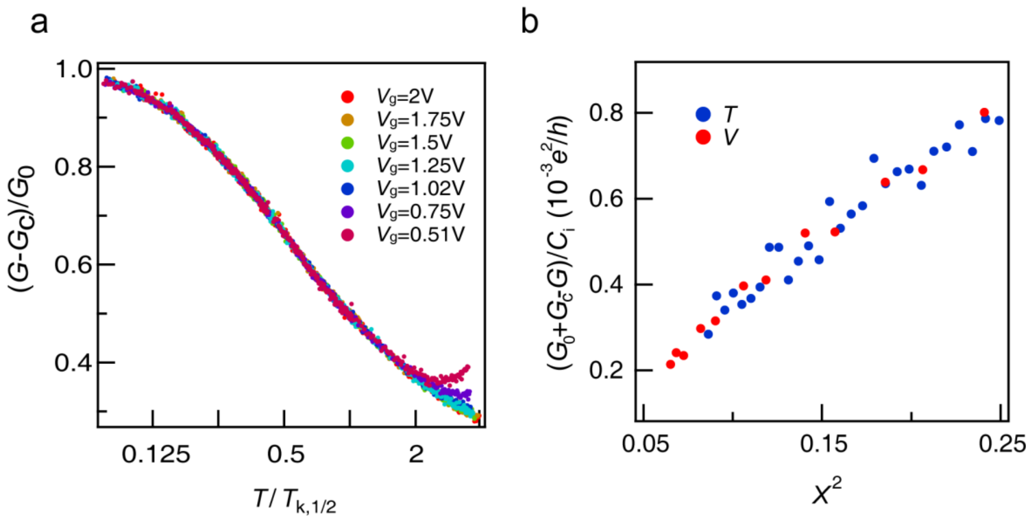

Supplementary Fig. 7 Universality of spin 1/2 Kondo effect. **a** The normalized zero bias conductance, $(G\text{-}G_c)/G_0$ for device 1 in region I at various gate voltages, shows a universal function of $T/T_{K,1/2}$.. **b** Plot of scaled low-energy differential conductance $(G_0+G_c\text{-}G)/C_i$(V_g=2V and C_i are the coefficients, i= V and T) against $\left(\frac{eV_{\mathrm{sd}}}{k_{\mathrm{B}}T_{\mathrm{K},1/2}}\right)^2$ and $\left(\frac{\pi T}{T_{\mathrm{K},1/2}}\right)^2$ (represented by X^2). The linear relation between conductance and X demonstrates a quadratic behavior for T and V. Here C_v=0.46, C_T =0.35 .

Supplementary References

1 Gobernadomitre, I., Aroca, R. & Desaja, J. A. Far-infrared spectra of monophthalocyanine and bisphthalocyanine derivatives *Spectroc. Acta Pt. A-Molec. Biomolec. Spectr.* **50**, 1243-1249 (1994).

2 Kobayashi, T. The far infrared spectra of phthalocyanine and its metal derivatives. *Spectrochimica Acta Part A: Molecular Spectroscopy* **26**, 1313-1322 (1970).

3 Sauvage, F. X., Debacker, M. G. & Stymne, B. An infrared study of the complexing ability of manganese phthalocyanine. *Spectroc. Acta Pt. A-Molec. Biomolec. Spectr.* **38**, 281-288 (1982).

4 Goldhaber-Gordon, D., Gores, J., Kastner, M. A., Shtrikman, H., Mahalu, D. & Meirav, U. From the Kondo regime to the mixed-valence regime in a single-electron transistor. *Phys. Rev. Lett.* **81**, 5225-5228 (1998).

5 Parks, J. J., Champagne, A. R., Costi, T. A., Shum, W. W., Pasupathy, A. N., Neuscamman, E., Flores-Torres, S., Cornaglia, P. S., Aligia, A. A., Balseiro, C. A., Chan, G. K., Abruna, H. D. & Ralph, D. C. Mechanical control of spin states in spin-1 molecules and the underscreened Kondo effect. *Science* **328**, 1370-1373 (2010).

6 Nagaoka, K., Jamneala, T., Grobis, M. & Crommie, M. F. Temperature dependence of a single Kondo impurity. *Phys. Rev. Lett.* **88**, 077205 (2002).
